# Trends in multimorbidity, complex multimorbidity and multiple functional limitations in the ageing population of England, 2002–2015

**DOI:** 10.1177/2235042X19872030

**Published:** 2019-09-04

**Authors:** Leo Singer, Mark Green, Francisco Rowe, Yoav Ben-Shlomo, Hill Kulu, Karyn Morrissey

**Affiliations:** 1 Department of Geography and Planning, University of Liverpool, Liverpool, UK; 2 Bristol Medical School, Population Health Sciences, University of Bristol, Bristol, UK; 3 School of Geography and Sustainable Development, University of St Andrews, Scotland, UK; 4 College of Medicine and Health, University of Exeter, Exeter, UK

**Keywords:** Multimorbidity, complex multimorbidity, functional limitations, trends, prevalence, ELSA, England, age, ageing, sex, socio-economic status, household wealth

## Abstract

This study aimed to estimate the prevalence of three measures of multimorbidity among people aged 50 years or older in England. Beside the basic measure of two or more diseases within a person, we added a measure of three or more affected body systems (complex multimorbidity) and a measure of 10 or more functional limitations. We found that the three health outcomes became more prevalent between 2002 and 2015. They were more common among females than males and were becoming more common among younger age groups. While in 2002, the prevalence of basic multimorbidity overcame 50% from the 70–74 age group upwards, in 2015 it crossed the same threshold in the 65–69 age group. The distribution of multimorbidity and multiple functional limitations were stratified by the amount of household wealth. Multiple functional limitations reflected the largest differences between the most and the least affluent groups (5.9-fold in 2014/2015), followed by the measure of complex multimorbidity (2.8-fold in 2014/2015) and basic multimorbidity (1.9-fold) in 2014/2015.While age acted as a levelling factor for the wealth differences in basic multimorbidity, it had no such effect on the two other outcomes. Our study observed social polarization among multimorbid ageing population in England where complex multimorbidity and multiple functional limitations increase faster and reflect stronger inequality than basic multimorbidity.

## Background

Multimorbidity (MM) when defined as the co-occurrence of two or more diseases within a person^1^ is rising globally.^2,3^ Its prevalence among people aged 65 years or older in England is projected to rise from 54% in 2015 to 67.8% in 2035.^4^ People will live longer but in worse health. The extra years lived with MM will lead to higher utilization of primary and secondary healthcare.^4^ The definition of MM as two or more diseases underpinning these statistics has been criticized for leading to prevalence estimates in the elderly population which are too high (55–98% between studies) to be able to predict patients with higher need.^5,6^ Practitioners need a measure of MM that can reflect the biology of ageing and identify elderly populations with higher healthcare needs.

Harrison et al.^7^ introduced the concept of complex multimorbidity (CMM), defined as ‘the co-occurrence of three or more chronic conditions affecting three or more different body systems within one person without an index chronic condition’.^7^ Compared to the basic definition of two or more conditions, the construct of CMM leads to lower prevalence estimates and it has been proposed that it might better identify patients with higher needs.^6,7^ We argue that CMM might also be better at reflecting the biology of ageing since it characterizes a simultaneous breakdown or dysfunction of several distinct pathologies or body systems.^8,9^ The affected body systems of people aged 65 or older were found as predictors of the total number of hospital stays and of the number of hospital admissions.^10^

The process of ageing manifests itself not just in the number of morbidities an individual has but also in physical functioning. A measure of multiple functional limitations (MFLs) was included as our third health outcome. Its purpose is to identify the impact of MM on the combined functioning of ageing people. Some conditions (e.g. high blood pressure) may have no effect on the physical functioning but others do, such as arthritis. MM predicts a decline in physical functioning among ageing people,^11,12^ which has implications for quality of life, need for healthcare, residential care and premature mortality.^11–13^ Measuring MFLs also responds to the fact that the proportion of old people with impairments and limitations in several body systems increases with age.^12,14^

Socio-economic status (SES) is a major determinant of health inequalities. Studies which explored the association between MM and SES focused on area deprivation,^15–17^ income,^18^ occupational status^19^ and education.^18,20^ Regardless of the type of measure, MM is more common among people with lower SES, even when controlling for age and sex. However, all of these studies focus on the simple definition of MM that may hide the nuances of relationships and the true underlying scale of inequalities.

Our study is the first population-level analysis that differentiates the prevalence of MM by complexity and degree of functional limitation as well as their variation by key modifying factors. The study has two aims: (1) to compare temporal trends in the prevalence of basic MM, CMM and MFLs in an ageing population in England and (2) to examine the variation in their prevalence by age, sex and SES.

## Methods

### Data and study population

We used data from the English Longitudinal Study of Ageing (ELSA) which is a panel study with a range of social, economic, psychological, cognitive and health data. It is based on a representative sample of people living in England aged 50+ years. It commenced in 2002 and is followed up every 2 years. The data used in this analysis was collected via personal interviews and the study response rate at wave 7 was 61%.^21^ The baseline sample consisted of 12,099 members. This analysis uses data from the core sample members who were recruited at either the first wave or at any of the refreshment samples at waves 3, 4, 6 and 7.^22^ The effects of clustering and stratification in a complex sample design such as ELSA were taken into account using wave-specific weights. The weights include a scaling factor to make sure that the original sample and refreshment samples are as equally proportional with respect to age as in the general population.^21^

### Measures of health

ELSA records data on a range of physical and mental health conditions. Twenty five of these variables were consistently recorded at each wave and are used to measure multiple health conditions in this study (Table 1). This includes the most common conditions among the elderly (diabetes, hypertension, stroke, cancer and depression), as found by a systematic literature review.^23^

**Table 1.** Health data used to measure basic multimorbidity, complex multimorbidity and multiple functional limitations.

	Morbidities	Body systems		Functional limitations
1	High blood pressure	1. Eye disorders		General mobility
2	Angina	1.1. Glaucoma	1	Walking 100 yards
3	Congested heart failure	1.2. Macular degeneration	2	Sitting for 2 h
4	Heart murmur	1.4. Cataracts	3	Getting up from chair
5	Abnormal heart rhythm	2. Circulatory disorders	4	Climbing several flights of stairs
6	Heart attack	2.1. High blood pressure	5	Climbing one flight of stairs
7	Diabetes	2.2. Angina	6	Stooping, kneeling or crouching
8	Stroke	2.3. Heart attack	7	Reaching arms above shoulders
9	Lung disease	2.4. Congestive heart failure	8	Pulling or pushing a chair
10	Asthma	2.5. Heart murmur	9	Lifting/carrying weights over 10 pounds
11	Arthritis	2.6. Abnormal heart rhythm	10	Picking up a 5p coin
12	Osteoporosis	2.7. Stroke		Activities of daily living
13	Cancer	3. Endocrine, nutritional and metabolic	11	Dressing, including putting on shoes and socks
14	Parkinson’s disease	3.1. Diabetic eye disease	12	Walking across a room
15	Dementia	3.2. Diabetes	13	Bathing or showering
16	Alzheimer’s disease	4. Musculoskeletal and connective system	14	Eating, such as cutting up your food
17	Hallucinations	4.1. Osteoporosis	15	Getting in or out of bed
18	Anxiety	4.2. Arthritis	16	Using the toilet, including getting up or down
19	Depression	5. Respiratory	17	Using a map to figure out how to get around
20	Emotional problems	5.1. Lung disease	18	Preparing a hot meal
21	Mood swings	5.2. Asthma	19	Shopping for groceries
22	Glaucoma	6. Neoplasms	20	Making telephone calls
23	Diabetic eye disease	6.1. Cancers	21	Taking medications
24	Macular degeneration	7. Nervous disorders	22	Doing work around the house or garden
25	Cataracts	7.1. Parkinson’s disease	23	Managing money (paying bills, track of expenses)
		7.2. Alzheimer’s disease		Symptoms
		7.3. Hallucinations	24	Difficulty walking 0.25 mile
		8. Mental and behavioural	25	Pain in general
		8.1. Anxiety	26	Problems with eyesight
		8.2. Depression	27	Problems with hearing
		8.3. Emotional problems	28	Balance on level surface
		8.4. Mood swings	29	Dizzy walking on level surface

Participants were asked whether they still had the condition diagnosed by a doctor that they had reported previously and if not whether they could report a new condition. We have grouped health data into three categories: individual morbidities, groups representing body systems and functional limitations (Table 1). Adapting Verbrugge and Jette’s disablement process framework,^24^ instances of impairment (dysfunction and abnormalities in body systems) and disability (difficulty with daily activities) were included within the category of ‘functional limitations’ (restrictions in basic physical and mental actions).

### Measure 1: Multimorbidity

We created a binary variable which identified people at each wave who had two or more morbidities as listed in Table 1. The list includes a few symptoms such as hallucinations which do not represent a condition but can be used as a proxy for schizophrenia or another condition (e.g. alcohol dependency).^25^ In a similar way, emotional problems and mood swings are used as indicators of either mild anxiety and depression or possibly manic depressive tendencies^26^ but the clinician has chosen not to use the more formal diagnostic label, for whatever reason. The information on whether an individual has or has not got a chronic disease was composed of the data fed forward from the previous wave of observation and from the information on the newly reported cases of disease.

### Measure 2: Complex multimorbidity

Following the definition of CMM by Harrison et al.,^7^ we identified individuals with three or more body systems affected by disease as having CMM. Body systems were defined and represented by the chapters of the International Classification of Diseases 10th Revision system (Table 1).

### Measure 3: Multiple functional limitations

The third health outcome was based on the combination of general mobility variables, activities of daily living (ADL) variables and data on symptoms of chronic conditions (Table 1). ADL is used to measure functional capacity and it concerns the abilities necessary for basic functioning, as well as functions necessary for living in a community.^27^ Most studies have explored prevalence and effects of either single impairments and functional limitations or their combinations in ADL or instrumental ADL, but we decided to examine their combined burden by summing all of them up including the symptoms. Difficulties with walking were captured with three distinct variables (having difficulty walking 0.25 mile, walking 100 yards and walking across a room) which, if combined, reflect the degree of severity. For example, a person who has got all three difficulties is more functionally limited than a person with only one of them. The total number of functional limitations per individual was summed up. Based on the exhaustive list of 29 limitations, the frequencies of MFLs were high, reflecting the older age of participants. To identify the participants with the highest level of disability we decided to set a cut-off point of 10 or more functional limitations within the same person.

## Covariates

### Age

Age was categorized into 5-year bands, from 50–54 up to 80–84 years of age. The age of persons aged 85 and older was collapsed in one category 85+ due to small sample size.

### Sex

Sex is an important determinant of health. Previous studies have shown that while women in most countries have a longer life expectancy than men, they are more likely to be affected by a number of chronic diseases.^5,28,29^

### Socio-economic status

SES was measured using quintiles of net total household wealth. Household wealth embodies access to financial resources accumulated during life and therefore reflects social status at later life.^30,31^ The net household wealth is defined as the sum of savings, investments, physical wealth and housing wealth after financial debt and mortgage debt have been subtracted. It is based on 22 distinct components of wealth and debt.^21^ The wealth intervals in £s between 2002 and 2015 are presented in Online Supplementary Material B, Table B.4. While the median value of households increased in 2002–2015 from £100,000 to £190,000, most change was due to the outlier values in the poorest and the richest quintiles.

### Statistical analysis

Descriptive analyses of the study population included summary statistics to explore general patterns. Data were weighted for non-response, stratification and clustering effects. The variation in the size of the age groups decreased over time for age groups of older people, but the pattern nevertheless justified the need for age standardization between waves (Online Supplementary Material A). The prevalence was standardized to the age distribution of the population at wave 1 in 2002, to allow for more robust comparison of trends over time. Standardization also helps our results to remain representative of national patterns improving their generalizability.

We have conducted repeated cross-sectional analyses of prevalence at a population level. Prevalence estimates were stratified by age groups, sex and wealth quintiles, to observe the distribution of outcomes by selected covariates. We then checked for consistency and interaction effects of Time × SES and Age × SES by merging the waves of measurement into a panel dataset. This allowed us to compare the estimates from cross-sectional analyses with two multilevel logistic regression models, taking into account temporal correlation within individuals. The results were plotted graphically using marginal effects at representative values. All analyses were conducted in Stata version 13.

## Results

### General characteristics of the study population

The general characteristics of the studied population are shown in Table 2. The number of participants varied between 11,391 (2002/2003) and 8249 (2014/2015). The median age in 2002/2003 was 64 (interquartile range (IQR) 56–73) and it increased to 67 years in 2014/2015 (IQR 61–75). The proportion of the oldest old people, aged 85 or more, was between 5.2% in 2002/2003 and 5.7% in 2014/2015. The proportion of women was higher than the proportion of men (53.1%) on average during the period 2002–2015.

**Table 2.** ELSA population distribution by age, sex and year.

	2002/2003	2004/2005	2006/2007	2008/2009	2010/2011	2012/2013	2014/2015
Age (years)	*n* (%, weighted)	*n* (%, weighted)	*n* (%, weighted)	*n* (%, weighted)	*n* (%, weighted)	*n* (%, weighted)	*n* (%, weighted)
50–54	1981 (19.4)	744 (9.6)	1388 (14.3)	1043 (13.3)	215 (3.1)	635 (17.7)	564 (19.5)
55–59	2185 (17.9)	1853 (21.4)	1658 (21)	1861 (22.7)	1753 (23.7)	1427 (17.8)	917 (16.7)
60–64	1688 (14.8)	1477 (16.1)	1421 (16.7)	2013 (17.7)	1976 (20.3)	1725 (16.7)	1499 (16.7)
65–69	1710 (13.6)	1397 (15)	1176 (13.7)	1497 (13.3)	1534 (15.6)	1726 (15.2)	1652 (15.3)
70–74	1471 (12.3)	1211 (12.7)	1130 (11.7)	1455 (11.5)	1389 (13)	1274 (11.1)	1281 (15.6)
75–79	1094 (10.2)	977 (11.2)	908 (9.8)	904 (9.4)	1025 (10.4)	1170 (9.1)	1134 (11.4)
80–84	806 (6.8)	698 (8.2)	623 (7.2)	609 (6.4)	646 (7.1)	642 (6.7)	657 (9.2)
85–100	456 (5.2)	423 (5.7)	506 (5.75)	513 (5.7)	552 (6.2)	570 (5.5)	545 (5.7)
Total	11,391	8780	8811	9896	9090	9169	8249
Male % (95% CI)	46.3 (45.7–47)	46.1 (45.4–46.8)	46.8 (46.1–47.6)	47 (46.2–47.8)	46.9 (46.1–47.7)	47.4 (46.5–48.4)	47.6 (46.4–48.7)
Female % (95%)	53.7 (53–54.3)	53.9 (53.2–54.6)	53.2 (52.4–53.9)	53 (52.2–53.7)	53.1 (52.3–53.9)	52.6 (51.6–53.5)	52.4 (51.3–53.6)
Age (median, IQR)	64 (56–73)	66 (58–74)	64 (57–74)	65 (58–73)	66 (60–74)	66 (60–75)	67 (61–75)

ELSA: English Longitudinal Study of Ageing; CI: confidence interval; IQR: interquartile range.

### Trends in the prevalence of our measures of MM

**Figure 1.**
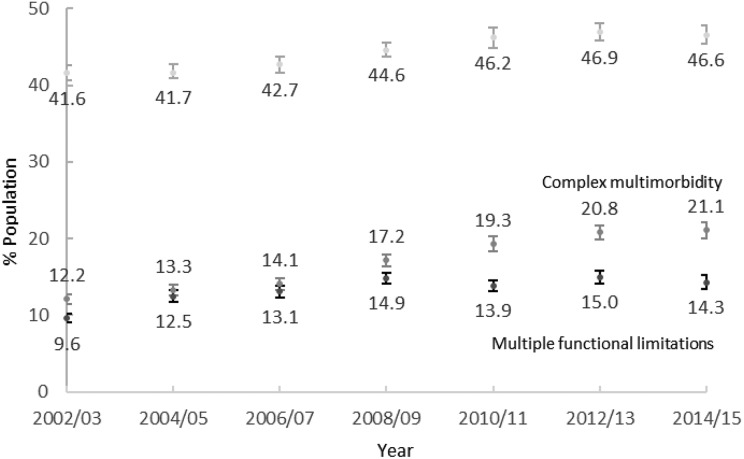
Age-standardized prevalence of basic multimorbidity, complex multimorbidity and multiple functional limitations for England, 2002–2015 (95% CIs). CI: confidence interval.

Figure 1 summarizes trends in the prevalence of basic MM, CMM and MFLs. The prevalence of MM grew from 41.6% in 2002/2003 to 46.6% in 2014/2015. The prevalence of CMM grew from 12.2% in 2002/2003 to 21.1% in 2014/2015. This is a larger change relative to the baseline estimate than the growth of basic MM. The prevalence of MFLs rose from 9.6% in 2002/2003 to 14.3% in 2014/2015 which is larger than the growth of basic MM. Given our knowledge of the nature of functional limitation as a consequence of MM,^11,12^ we would expect a larger relative change in this outcome than in either of the multimorbidities. Hence, we examined developments for each component subgroup (general mobility, ADLs and symptoms) separately and found similar flat trend for each of them (Online Supplementary Material E, Figure 7).

**Figure 2.**
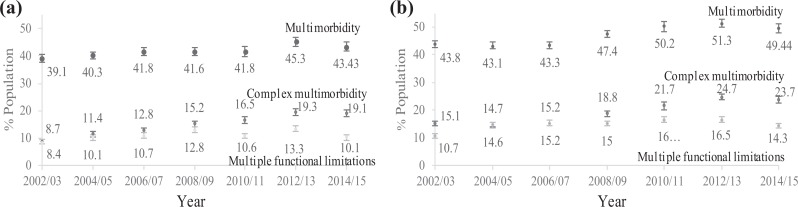
Age-standardized prevalence of basic multimorbidity, complex multimorbidity and multiple functional limitations by sex for England, 2002–2015 (95% CIs): (a) males and (b) females. CI: confidence interval.

Figure 2 shows the distribution of the three health outcomes by sex over time. The comparison between sexes shows that regardless of the measure of MM or specific time point, on average a higher proportion of women have MM than men. The difference in the change of prevalence between sexes over time was only marginal with the only exception being in CMM. The prevalence for males more than doubled at the end of the followed period while the prevalence of CMM for females grew only 1.6 times.

### Prevalence of the three measures of MM by age group

We next explored how the prevalence varied within age bands for each measure. The prevalence of both types of MM and of MFLs at each time point increased with age (see Online Supplementary Material B, Tables B.1–B.3). The difference in prevalence of MM between the youngest (aged 50–54) and the oldest group (aged 85+) ranged between threefold in wave 2012/2013 and fourfold in wave 2004/2005 (Online Supplementary Material B, Table B.1). The majority of participants were multimorbid when and after reaching the 70–75 age group. From 2012/2013, this threshold shifted to the 65–69 age band.

The difference in the prevalence of CMM between the youngest and the oldest group ranged between 4.6 times in 2010/2011 and 8.8 times in wave 2004/2005 (Online Supplementary Material B, Table B.2). The variation in prevalence levels by age is larger in the complex than basic MM. The difference in the prevalence of 10+ functional limitations between the youngest and the oldest group ranged between 3.9 times in wave 2010/2011 and 7.2 times in 2014/2015 (Online Supplementary Material B, Table B.3). Prevalence of both CMM and 10+ MFLs remained under 50% within each age group.

### Stratification of prevalence by SES

**Figure 3.**
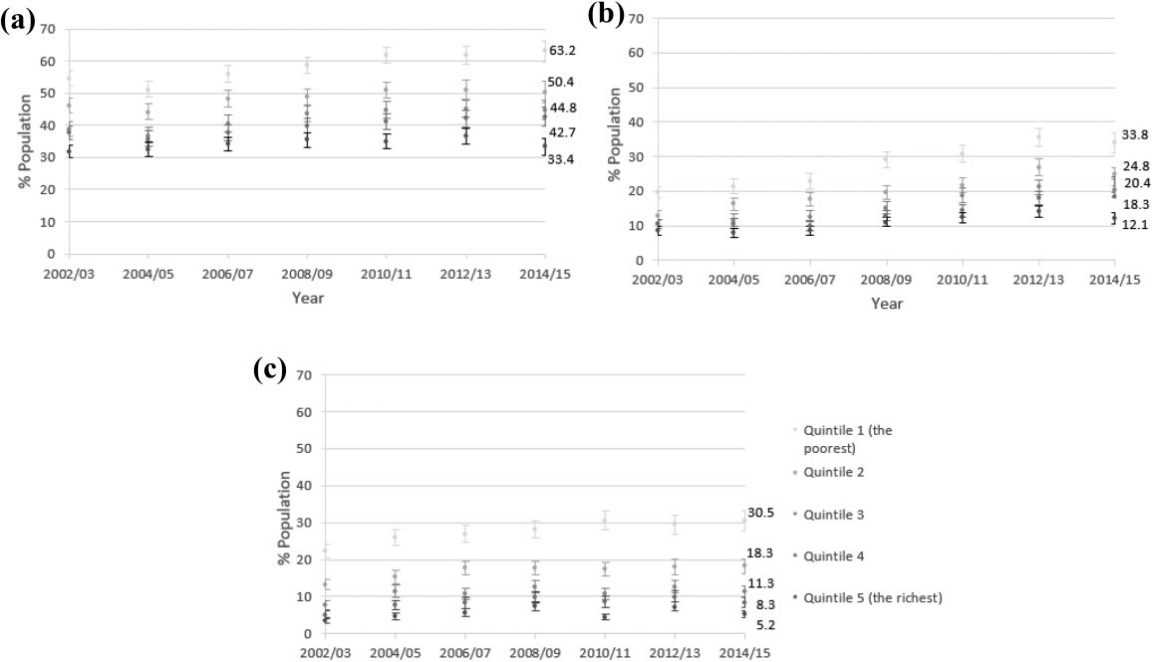
Age-standardized prevalence of (a) basic multimorbidity, (b) complex multimorbidity and (c) 10+ multiple functional limitations by quintiles of household wealth for England, 2002–2015 (95% CIs). CI: confidence interval.

Regardless of the outcome, clear differences between the socio-economic groups were observed (Figure 3). Prevalence of MM, CMM and MFLs was graded by each wealth quintile with people in the poorest quintile having the highest prevalence and people in the wealthiest quintile having the lowest. The measure of the MFLs captured the largest relative differences between the most and the least affluent groups (5.9-fold in 2014/2015), followed by the measure of CMM (2.8-fold in 2014/2015). The relative difference was the smallest for basic MM (1.9-fold) in 2014/2015. The interaction between time and household wealth was tested in a logistic marginal effects model and the results agreed with the stratified distribution of the prevalence (Online Supplementary Material C).

**Figure 4.**
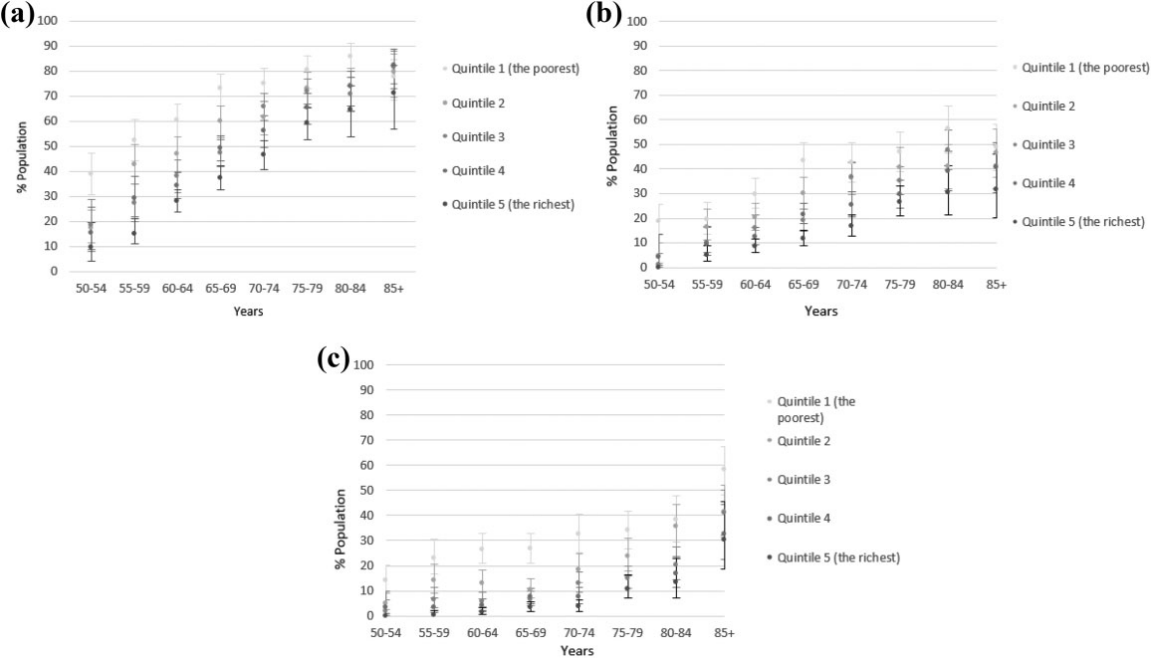
Prevalence of (a) basic multimorbidity, (b) complex multimorbidity and (c) 10+ multiple functional limitations by age band and wealth quintile for England, 2014/2015 (95% CIs). CI: confidence interval.

We further stratified each age band by quintiles of household wealth to observe differences in prevalence of our measures (Figure 4).To avoid data clutter, we report only results for the observation in 2014/2015. We found the largest variation in the 50–54 age group. The prevalence of basic MM in the poorest quintile was 4.1-times higher than in the richest quintile in the youngest age group. People aged 50–54 years in the poorest quintile had levels of MM equivalent to people 15–20 years older in the most affluent quintile. The prevalence of CMM and MFLs in the poorest category was 18.7-times and 14-times higher than in the wealthiest category in the youngest age group. People aged 50–54 years in the poorest quintile had levels of MM equivalent to people 20 years older (for CMM) and 30 years older (for MFLs) in the most affluent quintile.

The patterns in Figure 4 indicated that the effect of age on the prevalence estimates varies by SES. The interaction effect for the whole period 2002–2015 was further explored in a logistic regression model. The marginal effects (see Online Supplementary Material D, Figure 6) show changes in the probability of an outcome as the values of the household wealth variable change between quintiles. The additional effect of change in wealth quintile on the probability of having MM in 2014/2015 was the strongest in the lowest wealth quintile up to the age of 80–84. An overall pattern for all quintiles represents a socio-economic gradient up to the age of 75–80. For older age groups, the effects overlap and no pattern is discernible any more. The pattern changes for people with MFLs. The graded differences in effects between quintiles are more pronounced and they remain distinct even in the oldest age category. This confirms the distribution for 2014/2015 identified in Figure 4.

## Discussion

### Key results

Our study found that the prevalence of basic MM, CMM and MFLs in the ageing population of England increased between 2002/2003 and 2014/2015. We standardized our analysis to remove differences in age structure over time but in absolute terms, this increase will be even larger due to the ageing population. Also the addition of refreshment samples (age 50–53) at waves 3, 4, 6 and 7 has potentially resulted in an underestimation of the prevalence. The distribution of these health outcomes at population level was influenced by sex as they were more common among women than among men. Age was another determinant of the distribution. Our health outcomes were becoming more common in younger age groups during the observed period. The age when majority of an age group became multimorbid shifted from the 70–74 age group to the 65–69 age group (Online Supplementary Material B, Table B.1). Out of the three measures, the prevalence of CMM had the steepest growth, followed by MFLs and basic MM. Furthermore, the prevalence of MM, CMM and MFLs was socially stratified. People with less household wealth had higher levels of multiple health problems than people from the more affluent wealth quintiles. The disparity in wealth was larger for CMM and functional limitations than for basic MM.

We also discovered that SES and age mutually interacted. The differences in the prevalence of basic MM between the wealth quintiles were the largest in the youngest age group and they narrowed down as people aged (Figure 4). The differences in the prevalence of CMM and especially MFLs between the poorest and wealthiest quintile remained large for all age groups (Figure 4).

The pattern of health inequality based on cross-sectional stratification analyses in Figures 3 and 4 was confirmed after data were reshaped into a panel design where time interacted with wealth (Online Supplementary Material C, Figure 5) and age interacted with wealth (Online Supplementary Material D, Figure 6).

### Interpretation

The rising prevalence of MM consistent across three different conceptualizations between 2002/2003 and 2014/2015 supports projections of a growing trend.^4^ Prevalence in general is shaped by both the rate at which new cases are occurring and the average duration of disease. Our analysis was a repeated cross-sectional and as such it examined neither the incidence nor the duration of MM and cannot quantify their relative contribution to the increased prevalence.

Household wealth, an indicator of SES, was negatively associated with MM and MFLs. This is consistent with previous studies reporting socio-economic gradient in MM.^15–20,32^ Our study observed that the gap between the wealth quintiles was larger for participants with CMM and the largest for people with 10 or more functional limitations. Lack of household wealth was related to higher complexity of MM and corresponding limitations and vice versa. This is consistent with the findings of a study examining growth in functional limitations and socio-economic factors.^33^ It seems plausible that this gradient in complexity might be explained by problems with the self-management of MM. Patients whose everyday lives are overwhelmed by acute social problems are less able to manage the complex treatment burden and find adequate social support.^34^ This would suggest that the true impact of inequalities is underestimated if MM is defined as the presence of two or more conditions or, similarly, if the cut-off measure for number of functional limitations is set too low.

Ageing with MM and functional limitation was differentiated by SES. We observed an excess of multiple health problems in the youngest age cohort with lowest SES. People aged 50–54 years in the poorest quintile had levels of CMM comparable to those 20 years older in the most affluent quintile and level of functional limitations comparable to those 30 years older in the top wealth quintile. This suggests an earlier onset of MM, and especially of CMM and MFLs, for people with lower SES. Earlier origins of basic MM in Scotland were observed by Barnett et al.^15^ The differences in the prevalence between the wealth quintiles were the largest in the youngest age group but they narrowed down as people aged. Similar levelling effects of ageing on basic MM prevalence have been reported previously.^15^ The differences in the prevalence of CMM and especially MFLs between the poorest and wealthiest quintile remained large for all age groups. This suggests that accumulated financial resources at older age can act as a protective factor against increased disease complexity. One pathway in which this accumulated financial resources may protect against increased disease complexity is via financial advantage translating into an actual healthy behaviour. For example, Link and Phelan postulated that individuals from higher social class backgrounds are capable to use resources such as power, money, knowledge, prestige or social contacts to either protect themselves from the health risks or compensate for their existing disease burden.^35^

### Limitations

Our exploratory study focused on the assessment of the burden of MM, CMM and MFLs at the population level. Using a repeated cross-sectional design does not allow any explanatory inferences to be drawn regarding individual trends or causal relationships between covariates and outcome variables.

The estimates of prevalence might be underestimated as they are based on self-reported information on health problems. A previous study found that prevalence based on self-reports was lower than if data were obtained from medical examinations.^36^ A combination of data sources was suggested as the best way of providing the most reliable results.^6^

This study could be expanded if we had shown an association between the two MM measures and the measure of MFLs. Such an analysis might be interesting especially as both CMM and MFLs represent problems affecting multiple body systems.

## Conclusion

To the best of our knowledge, this article is the first study to examine trends in the prevalence of MM as measured through three types of conceptualizations of MM. We uncovered processes of clear polarization within the ageing population of England. Alongside stable proportion of people who were free of any chronic disease and declining proportion of those with one disease, we observed that the increase in complexity overtakes the rise in basic MM and MFLs. Another axis of differentiation is by SES where the higher household wealth is related to lower prevalence. At the same time, this process introduces health inequality within age groups. CMM and MFLs are increasing faster and capture stronger inequality than the measure of basic MM. Using different measures of MM can contribute to identify population groups with higher healthcare needs and to a better allocation of healthcare resources. Reporting the patterns of body systems affected by chronic conditions may help healthcare planners identify services which should be co-located, for an optimal care of these patients.^37^ The CMM measure would also allow identification of patients who may need help in coordinating care between multiple healthcare providers.

Policies aiming to prevent and reduce the growth in MM should be approaching older population as diverse and take into account the multiple polarizations we have described. It would be meaningful to focus the preventive efforts to younger age groups where social inequality appears to be more intertwined with chronic complexity and functional limitation than in older age. The contribution of these younger cohorts as they age into the older population, along with growing numbers of the very old, could significantly increase the health and social care costs in future.

## Supplemental material

Supplementary_material_v2 - Trends in multimorbidity, complex multimorbidity and multiple functional limitations in the ageing population of England, 2002–2015 Click here for additional data file. Supplementary_material_v2 for Trends in multimorbidity, complex multimorbidity and multiple functional limitations in the ageing population of England, 2002–2015 by Leo Singer, Mark Green, Francisco Rowe, Yoav Ben-Shlomo, Hill Kulu and Karyn Morrissey in Journal of Comorbidity

## References

[1] Van den Akker M Buntinx F Metsemakers JFM, et al. Multimorbidity in general practice: prevalence, incidence and determinants of co-occurring chronic and recurrent diseases. J Clin Epidemiol 1998; 51(5): 367–370. 9619963 10.1016/s0895-4356(97)00306-5

[2] Garin N Koyanagi A Chatterji S, et al. Global multimorbidity patterns: a cross-sectional, population-based, multi-country study. J Gerontol A Biol Sci Med Sci 2015; 71(2): 205–214. 26419978 10.1093/gerona/glv128 PMC5864156

[3] The Academy of Medical Sciences. Global burden of multiple serious illnesses must be urgently addressed. https://acmedsci.ac.uk/more/news/global-burden-of-multiple-serious-illnesses-must-be-urgently-addressed (2018, accessed 12 June 2018).

[4] Kingston A Robinson L Booth H, et al. Projections of multi-morbidity in the older population in England to 2035: estimates from the population ageing and care simulation (PACSim) model. Age Ageing 2018; 47(3): 374–380. 29370339 10.1093/ageing/afx201 PMC5920286

[5] Marengoni A Angleman S Melis R, et al. Aging with multimorbidity: a systematic review of the literature. Ageing Res Rev 2011; 10: 430–439. 21402176 10.1016/j.arr.2011.03.003

[6] Fortin M Stewart M Poitras ME, et al. A systematic review of prevalence studies on multimorbidity: toward a more uniform methodology. Ann Fam Med 2012; 10(2): 142–151. 22412006 10.1370/afm.1337 PMC3315131

[7] Harrison C Britt H Miller G, et al. Examining different measures of multimorbidity, using a large prospective cross-sectional study in Australian general practice. BMJ Open 2014; 4(7): e004694. 10.1136/bmjopen-2013-004694 PMC4120329 25015470

[8] Fabbri E Zoli M Gonzalez-Freire M, et al. Aging and multimorbidity: new tasks, priorities and frontiers for integrated gerontological and clinical research. J Am Med Dir Assoc 2015; 16(8): 640–647. 25958334 10.1016/j.jamda.2015.03.013 PMC5125299

[9] Cesari M Pérez-Zepeda MU Marzetti E, et al. Frailty and multimorbidity: different ways of thinking about geriatrics. J Am Med Dir Assoc 2017; 18(4): 361–364. 28279606 10.1016/j.jamda.2016.12.086

[10] Condelius A Edberg AK Jakobsson U, et al. Hospital admissions among people 65+ related to multimorbidity, municipal and outpatient care. Arch Gerontol Geriatr 2008; 46(1): 41–55. 17403548 10.1016/j.archger.2007.02.005

[11] Ryan A Wallace E O’Hara P, et al. Multimorbidity and functional decline in community-dwelling adults: a systematic review. Health Qual Life Outcomes 2015; 13: 168. 26467295 10.1186/s12955-015-0355-9 PMC4606907

[12] Jindai K Nielson CM Vorderstrasse BA, et al. Multimorbidity and functional limitations among adults 65 or older, NHANES 2005-2012. Prev Chronic Dis 2016; 13: E151. 27809419 10.5888/pcd13.160174 PMC5094859

[13] Sibbritt DW Byles JE Regan C Factors associated with decline in physical functional health in a cohort of older women. Age Ageing 2007; 36(4): 382–388. 17383985 10.1093/ageing/afm017

[14] Burden of Disease Network Project. Disability in old age: final report. https://ju.se/download/18.3783220012d8f123ca58000115/1520578695703/DISABILITY%20IN%20OLD%20AGE.pdf (2004, accessed 12 November 2018).

[15] Barnett K Mercer SW Norbury M, et al. Epidemiology of multimorbidity and implications for health care, research, and medical education: a cross-sectional study. Lancet 2012; 380(9836): 37–43. 22579043 10.1016/S0140-6736(12)60240-2

[16] McLean G The influence of socioeconomic deprivation on multimorbidity at different ages: a cross-sectional study. Br J Gen Pract 2014; 64(624): e440–e447. 24982497 10.3399/bjgp14X680545 PMC4073730

[17] Morrisssey K Espuny F Williamson P A multinomial model for comorbidity in England of long-standing cardiovascular diease, diabetes and obesity. Health Soc Care Community 2015; 24(6): 717–727. 25924692 10.1111/hsc.12251

[18] Agborsangaya CB Lau D Lahtinen M Cooke T, et al. Multimorbidity prevalence and patterns across socioeconomic determinants: a cross-sectional survey. BMC Public Health 2012; 12: 201. 22429338 10.1186/1471-2458-12-201 PMC3353224

[19] Van den Akker M Buntinx F Metsemakers JFM, et al. Marginal impact of psychosocial factors on multimorbidity: results of an explorative nested case–control study. Soc Sci Med 2000; 50(11): 1679–1693. 10795973 10.1016/s0277-9536(99)00408-6

[20] Schiøtz ML Stockmarr A Høst D, et al. Social disparities in the prevalence of multimorbidity – a register-based population study. BMC Public Health 2017; 17: 422. 28486983 10.1186/s12889-017-4314-8 PMC5424300

[21] Banks J Blake M Clemens S, et al. English longitudinal study of ageing: waves 0-8, 1998-2017. [data collection], 25th ed UK Data Service. SN: 5050. DOI: 10.5255/UKDA-SN-5050-13.

[22] Steptoe A Breeze E Banks J, et al. Cohort profile: the English longitudinal study of ageing. Int J Epidemiol 2013; 42(6): 1640–1648. 23143611 10.1093/ije/dys168 PMC3900867

[23] Sinnige J Braspenning J Schellevis F, et al. The prevalence of disease clusters in older adults with multiple chronic diseases – a systematic literature review. PLoS One 2013; 8(11): e79641. 24244534 10.1371/journal.pone.0079641 PMC3823581

[24] Verbrugge LM Jette AM The disablement process. Soc Sci Med 1994; 38(1): 1–14. 8146699 10.1016/0277-9536(94)90294-1

[25] Chaudhury S Hallucinations: clinical aspects and management. Indust Psychiat J 2010; 19(1): 5–12. 10.4103/0972-6748.77625 PMC3105559 21694785

[26] Valiengo L Stella F Forlenza OV Mood disorders in the elderly: prevalence, functional impact, and management challenges. Neuropsych Dis Treat 2016; 12: 2105–2114. 10.2147/NDT.S94643 PMC5003566 27601905

[27] Chatterji S Byles J Cutler D, et al. Health, functioning, and disability in older adults—present status and future implications. Lancet 2015; 385: 563–575. 25468158 10.1016/S0140-6736(14)61462-8 PMC4882096

[28] Abad-Díez JM Calderón-Larrañaga A Poncel-Falcó A, et al. Age and gender differences in the prevalence and patterns of multimorbidity in the older population. BMC Geriatr 2014; 14: 75. 24934411 10.1186/1471-2318-14-75 PMC4070347

[29] Agur K McLean G Hunt K, et al. How does sex influence multimorbidity? Secondary analysis of a large nationally representative dataset. Int J Environ Res Public Health 2016; 13(4): 391. 27043599 10.3390/ijerph13040391 PMC4847053

[30] Demakakos P Biddulph JP Bobak M, et al. Wealth and mortality at older ages: a prospective cohort study. J Epidemiol Community Health 2015; 70(4): 346–353. 26511887 10.1136/jech-2015-206173 PMC4819652

[31] Nazroo J Class and health inequality in later life: patterns, mechanisms and implications for policy. Int J Environ Res Public Health 2017; 14(12): 1533. 10.3390/ijerph14121533 PMC5750951 29292775

[32] Charlton J Rudisill C Bhattarai N, et al. Impact of deprivation on occurrence, outcomes and health care costs of people with multiple morbidity. J Health Serv Res Policy 2013; 18(4): 215–223. 23945679 10.1177/1355819613493772 PMC3808175

[33] Calderón-Larrañaga A Santoni G Wang HX, et al. Rapidly developing multimorbidity and disability in older adults: does social background matter? J Intern Med 2018; 283(5): 489–499. 29415323 10.1111/joim.12739

[34] O’Brien R Wyke S Watt GCM The ‘everyday work’ of living with multimorbidity in socioeconomically deprived areas of Scotland. J Comorb 2014; 4: 1–10. 29090148 10.15256/joc.2014.4.32 PMC5556407

[35] Link B Phelan J Social conditions as fundamental causes of disease. J Health Soc Behav 1995; 80–94. http://www.jstor.org/stable/2626958 (accessed 10 December 2018). 7560851

[36] Schramm M Frijters D Van de Lisdonk FH, et al. Setting and registry characteristics affect the prevalence and nature of multimorbidity. J Clin Epidemiol 2008; 61(11): 1104–1112. 18538993 10.1016/j.jclinepi.2007.11.021

[37] Harrison C Henderson J Miller G, et al. The prevalence of complex multimorbidity in Australia. Aust N Z J Public Health 2016; 40: 239–244. 27027989 10.1111/1753-6405.12509

