# Supplementary material for: Trends in multimorbidity, complex multimorbidity and multiple functional limitations in the ageing population of England, 2002–2015
Source: J Comorb. 2019 Sep 4;9:2235042X19872030. doi: 10.1177/2235042X19872030 (PMC6727093; doi:10.1177/2235042X19872030)
Supplement: Supplementary_material_v2 - Trends in multimorbidity, complex multimorbidity and multiple functional limitations in the ageing population of England, 2002–2015 [file Supplementary_material_v2.pdf]

Supplementary material A.

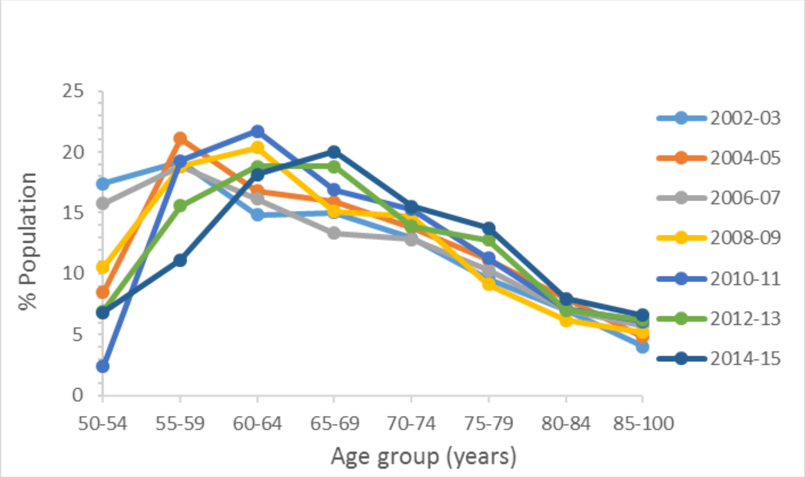

Table A.1 Age structure 2002-2015

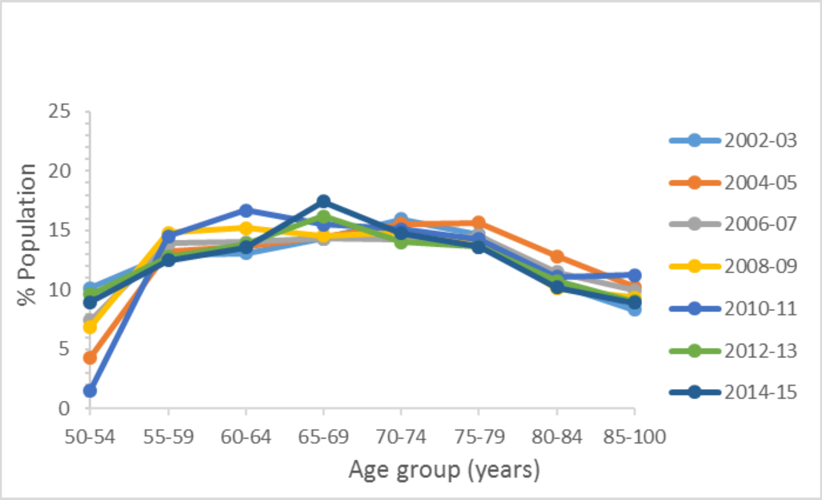

Table A.2 Distribution of multimorbidity by age group and time

Supplementary material B.

Table B.1 Prevalence of MM by age

	2002/03		2004/05		2006/07		2008/09		2010/11		2012/13		2014/15
Age (years)	n*	Prevalence (95 % CI)	n*	Prevalence (95 % CI)	n*	Prevalence (95 % CI)	n*	Prevalence (95 % CI)	n*	Prevalence (95 % CI)	n*	Prevalence (95 % CI)	n*	Prevalence (95 % CI)
50-54	481	21.8 (19.9-23.8)	158	18.8 (16.1-21.7)	279	22.5 (20.3-24.9)	303	23.3 (20.7-26)	69	24.7 (19.4-30.9)	422	26.3 (22.8-30.1)	375	23.6 (20.1-27.5)
55-59	616	30.3 (28.3-32.4)	485	25.8 (23.7-28)	523	28.9 (26.7-31.2)	652	29.3 (27.1-31.5)	645	30.3 (28.1-32.7)	563	35 (32.1-37.9)	521	38.3 (34.8-41.8)
60-64	619	36.8 (34.3-39.3)	501	35.5 (33-38)	528	36.5 (33.9-39.2)	668	38.5 (36.3-40.8)	743	40.7 (38.4-43)	611	40.3 (37.9-42.8)	568	45.6 (43-48.3)
65-69	678	43.8 (41.5-46.2)	530	40.3 (37.7-42.9)	538	45.4 (42.5-48.4)	640	49.2 (46.6-51.8)	689	49.3 (46.6-51.9)	713	51.8 (49.3-54.3)	729	57.3 (54.9-59.8)
70-74	755	54 (51.4-56.7)	570	50.9 (48.1-53.8)	534	52.9 (49.8-55.9)	652	57.8 (55.1-60.4)	671	57.3 (54.5-60)	617	61.3 (58.5-64)	616	66.2 (63.4-68.9)
75-79	696	59.8 (56.8-62.7)	574	58.3 (55.2-61.4)	548	64.6 (61.3-67.8)	623	67.4 (64.1-70.5)	634	67.7 (64.7-70.6)	600	72.4 (69.6-75)	570	75.8 (73.1-78.4)
80-84	496	64.3 (60.8-67.6)	471	65 (61.3-68.5)	433	69.6 (65.7-73.3)	445	70.7 (66.6-74.5)	493	77.3 (73.6-80.5)	474	78 (74.5-81.1)	426	79.2 (75.6-82.3)
85-100	396	68.9 (64.2-73.1)	376	74.7 (70-78.9)	375	75.3 (70.5-79.6)	412	74.1 (69.6-78.1)	501	82.8 (79-86.1)	397	77.7 (73.6-81.3)	372	80.2 (76.1-83.7)
Total	4,739	41.6 (40.6-42.6)	3,664	41.7 (40.6-42.9)	3,759	43.4 (42.3-44.5)	4,396	44.8 (43.8-45.9)	4,446	49.5 (48.4-50.6)	4,398	48.5 (47.3-49.8)	4,178	51.2 (49.9-52.6)
Mean age (SD)		68.9 (10.5)		70.7 (10.3)		70.1 (11.1)		69.8 (10.3)		70.8 (9.5)		70.8 (9.6)		71.4 (9.2)
Median age (IQR)		69 (60-77)		71 (62-78)		70 (61-78)		69 (62-77)		70 (63-78)		71 (64-78)		71 (65-78)

* Number of persons with MM

Table B.2 Prevalence of CMM by age

	2002/03		2004/05		2006/07		2008/09		2010/11		2012/13		2014/15	
Age (years)	n *	Prevalence (95 % CI)	n *	Prevalence (95 % CI)	n *	Prevalence (95 % CI)	n *	Prevalence (95 % CI)	n *	Prevalence (95 % CI)	n *	Prevalence (95 % CI)	n *	Prevalence (95 % CI)
50-54	90	4.1 (3.3-5.1)	32	3.8 (2.6-5.5)	77	6.2 (5-7.6)	77	5.9 (4.6-7.5)	25	8.9 (5.8-13.3)	131	8.2 (6.2-10.8)	113	7.1 (5.2-9.6)
55-59	122	6 (5.1-7)	109	5.7 (4.8-6.9)	144	8 (6.7-9.5)	196	8.8 (7.5-10.2)	180	8.5 (7.2-9.9)	230	14.3 (12.3-16.5)	163	12 (9.7-14.6)
60-64	142	8.4 (7.2-9.9)	140	9.9 (8.4-11.6)	164	11.4 (9.8-13.2)	240	13.8 (12.3-15.5)	261	14.3 (12.7-16)	260	17.2 (15.4-19.2)	200	16.1 (14.2-18.1)
65-69	168	10.8 (9.4-12.4)	182	13.9 (12.1-15.8)	158	13.4 (11.5-15.5)	240	18.4 (16.4-20.6)	287	20.6 (18.5-22.8)	325	23.6 (21.6-25.8)	293	24 (20.9-25.4)
70-74	233	16.7 (14.8-18.7)	208	18.5 (16.4-20.9)	184	18.2 (15.9-20.8)	265	23.5 (21.3-25.9)	298	25.4 (23-28)	302	30 (27.4-32.7)	282	30.3 (27.7-33.1)
75-79	245	21 (18.7-23.6)	224	22.8 (20.2-25.6)	195	23 (20.3-25.9)	288	31.1 (27.9-35.7)	309	33 (30-36.1)	325	39.2 (36.3-42.2)	266	35.5 (32.6-38.5)
80-84	223	28.9 (25.9-32)	210	29 (25.7-32.6)	176	28.3 (24.7-32.1)	199	31.6 (28.1-34.3)	255	40 (36-44)	275	45.2 (41.2-49.2)	236	43.8 (39.8-47.9)
85-100	178	31 (26.6-35.7)	168	33.4 (28.8-38.4)	151	30.4 (26.3-34.9)	194	34.9 (30.5-39.5)	250	41.3 (36.9-45.7)	214	41.9 (37.4-46.5)	203	43.6 (39.1-48.3)
Total	1400	12.3 (11.7-12.9)	1,272	14.5 (13.7-15.3)	1,250	14.4 (13.7-15.2)	1,698	17.3 (16.5-18.1)	1,865	20.8 (19.9-21.7)	2,062	22.8 (21.8-23.7)	1,756	21.5 (20.5-22.6)
Mean age (SD)		71.9 (10.6)		73 (9.9)		71.6 (11.2)		71.5 (10.2)		72.5 (9.4)		72 (9.5)		73 (9.1)
Median age (IQR)		72 (64-80)		74 (66-81)		72 (63-80)		71 (63-80)		73 (65-80)		72 (65-79)		73 (66-80)

* Number of persons with CMM

Table B.3 Prevalence of 10+MFLs by age

		2002/03		2004/05		2006/07		2008/09		2010/11		2012/13		2014/15
Age (years)	n*	Prevalence (95 % CI)	n*	Prevalence (95 % CI)	n*	Prevalence (95 % CI)	n*	Prevalence (95 % CI)	n*	Prevalence (95 % CI)	n*	Prevalence (95 % CI)	n*	Prevalence (95 % CI)
50-54	104	4.7 (3.9-5.7)	55	6.6 (4.9-8.7)	83	6.7 (5.5-8.2)	128	9.8 (8.1-11.9)	30	10.8 (7.3-15.6)	157	9.8 (7.5-12.6)	110	6.9 (5.1-9.3)
55-59	149	7.3 (6.3-8.5)	150	7.9 (6.8-9.3)	157	9.2 (7.8-10.8)	200	10.2 (8.8-11.7)	189	8.9 (7.5-10.5)	213	13.2 (11.3-15.4)	138	10.1 (8.1-12.5)
60-64	136	8.1 (6.8-9.5)	153	10.9 (9.4-12.6)	165	11.4 (9.8-13.3)	228	11.5 (10.1-13.2)	160	8.8 (7.5-10.2)	174	11.5 (10-13.2)	129	10.4 (8.8-12.2)
65-69	138	8.8 (7.6-10.3)	124	9.4 (8-11.1)	157	12.1 (10.3-14.1)	179	13.8 (12.1-15.7)	174	12.5 (10.7-14.4)	184	13.4 (11.7-15.2)	141	11.1 (9.5-12.9)
70-74	145	10.3 (8.8-12.1)	149	13.4 (11.5-15.5)	147	14.5 (12.5-16.8)	175	15.5 (13.6-17.6)	176	15 (13.1-17.2)	160	15.9 (13.8-18.2)	139	15 (12.9-17.3)
75-79	160	13.7 (11.7-16)	162	16.4 (14.1-19)	157	18.6 (16-21.4)	175	20.9 (18.3-23.9)	183	19.6 (17.2-22.3)	153	18.5 (16.2-21)	158	21 (18.5-23.8)
80-84	147	19 (16.4-21.9)	192	26.6 (23.3-30.1)	162	26 (22.4-29.8)	193	27.9 (24.2-31.9)	178	28 (24.4-31.8)	158	26 (22.5-29.9)	163	30.3 (26.7-34.2)
85-100	191	33.2 (28.7-37.9)	221	43.9 (38.8-49)	216	43.4 (38.6-48.3)	218	39.1 (34.5-44)	254	42 (37.6-46.7)	206	40.2 (35.8-44.8)	231	49.8 (45-54.6)
Total	1169	10.3 (9.6-10.9)	1,207	13.7 (12.9-14.6)	1,240	14.3 (13.5-15.1)	1496	15.3 (14.5-16.1)	1,346	15 (14.2-15.8)	1,405	15.5 (14.7-16.4)	1,210	14.8 (14-15.7)
Mean age (SD)		70.7 (12)		72.6 (11.8)		72.3 (12.6)		71.2 (12.1)		73.2 (10.6)		72 (11)		74.3 (10.6)
Median age (IQR)		70 (61-80)		73 (62-81)		72 (62-82)		70 (61-80)		73 (64-82)		72 (63-81)		75 (66-83)

* Number of persons with 10+ functional limitations

Table B.4 Household wealth bands in £s by year

Household wealth (£s i	2002/03		2004/05		2006/07		2008/09		2010/11		2012/13		2014/15	
	Lower bound	Upper bound	Lower bound	Upper bound	Lower bound	Upper bound	Lower bound	Upper bound	Lower bound	Upper bound	Lower bound	Upper bound	Lower bound	Upper bound
Quintile 1	-108,000	0	-130	27,5	-160	37,5	-450	56	-80	65	-20	65	-220	70
Quintile 2	3,000	75	29,5	129	37,5	150	56,5	150	66	150	66	150	71,1	160
Quintile 3	75,250	125	129,3	185	151	200	150,5	200	150,5	208	151	210	161	240
Quintile 4	126,000	200	185,4	265	201	300	200,2	300	208,7	300	211	320	241	350
Quintile 5	200,500	2,486	266,7	2,000	301	4,440	301	3,500	304	4,000	321	4,500	351	4,000
Median (IQR*)	100 (33-180)		155 (75-245)		175 (89-261)		175 (91-265)		180 (100-285)		180 (95-290)		190 (100-310)	
* Interquartile range														

Supplementary material C.

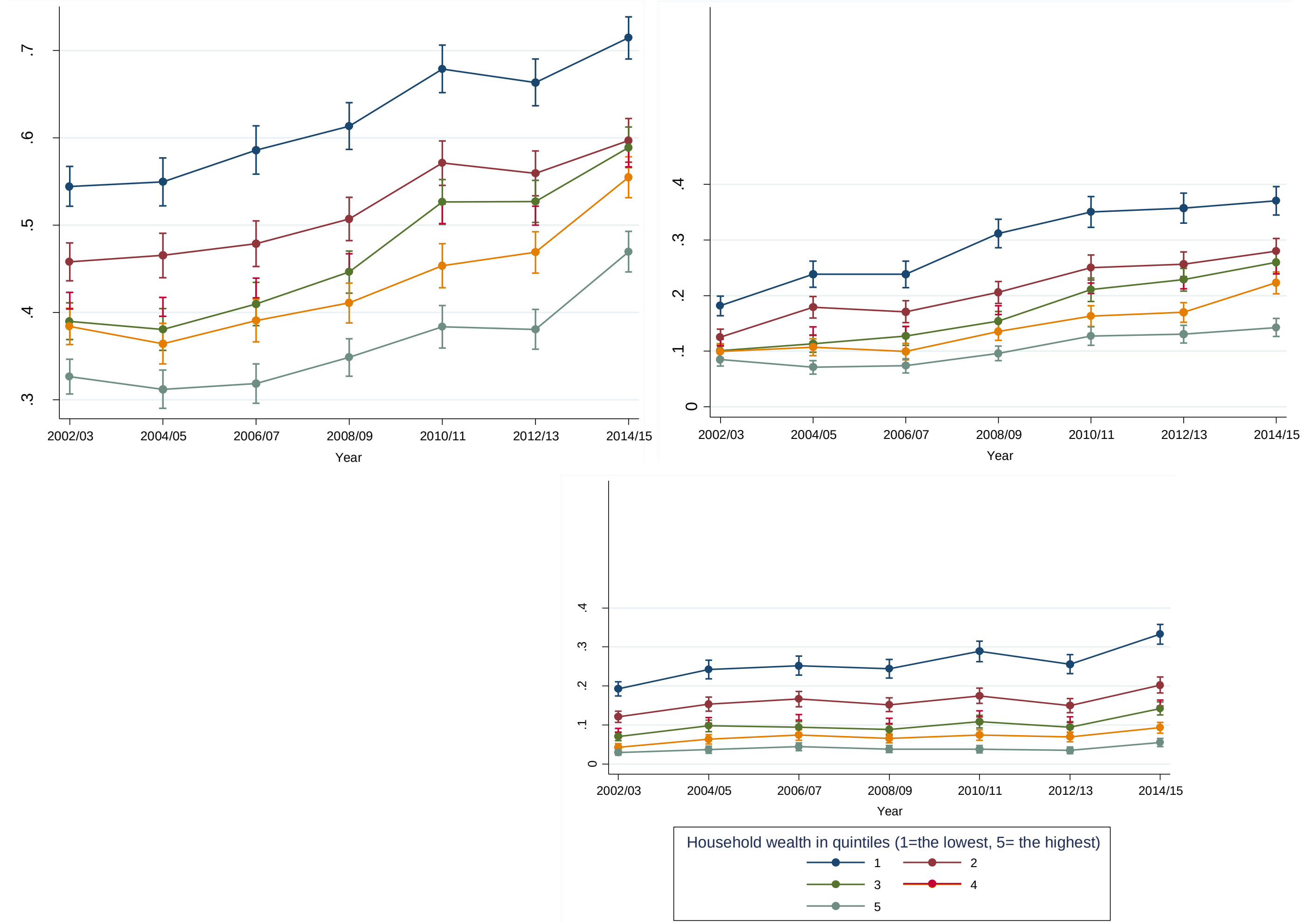

Figure 5. Marginal interaction effects of time and wealth on the probability of multimorbidity, complex multimorbidity and multiple functional limitations (95% CIs) in the period 2002-2015.

Supplementary material D.

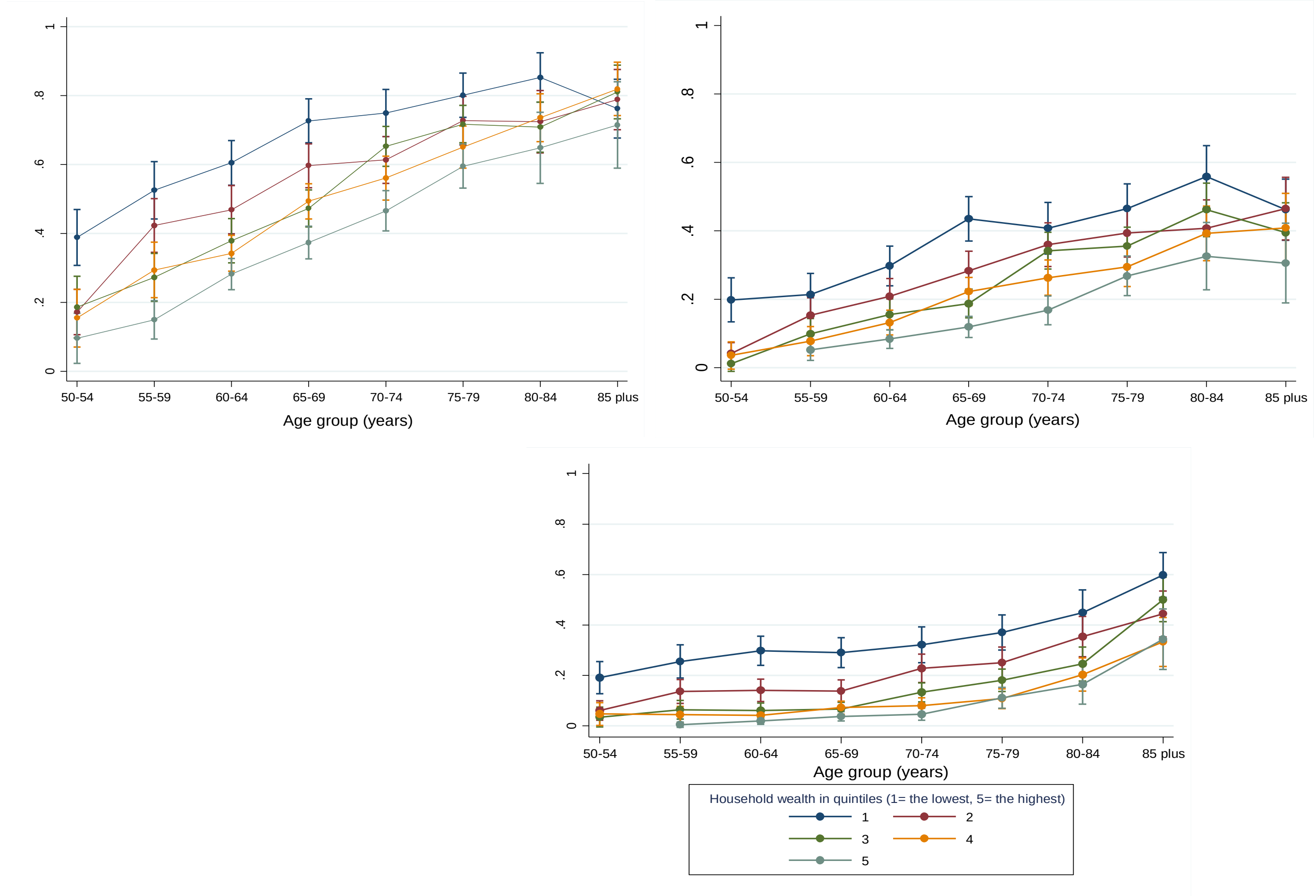

Figure 6. Marginal interaction effects of age and wealth on the probability of multimorbidity, complex multimorbidity and multiple functional limitations (95% CIs) in 2014/15.

Supplementary material E.

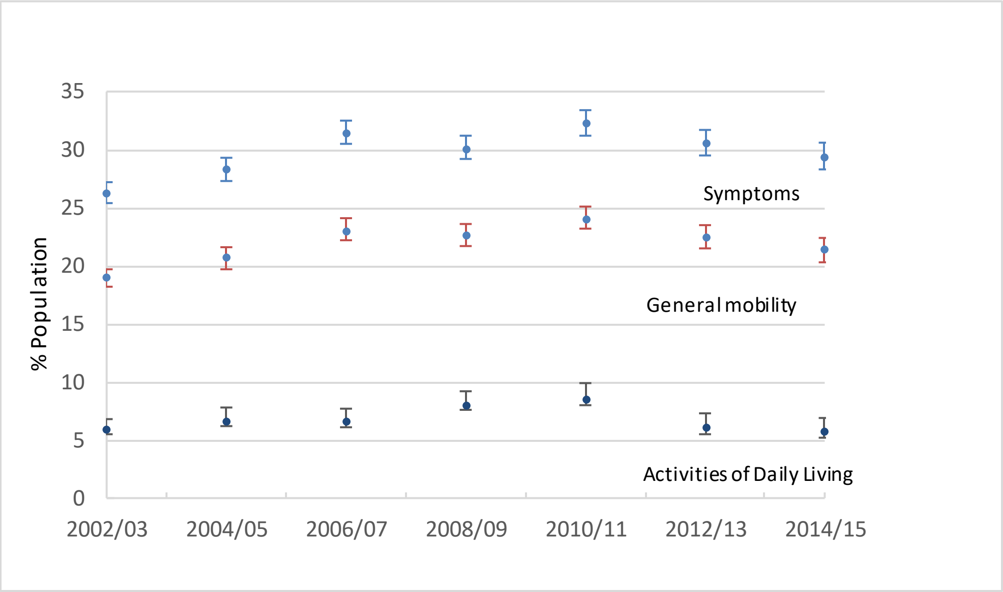

Figure 7. Prevalence of functional limitations in Activities of Daily Living, general mobility and symptoms for England in 2002-15 (95% CIs).
